# Correlates of Work-Study Conflict among International Students in Australia: A Multivariate Analysis

**DOI:** 10.3390/ijerph16152695

**Published:** 2019-07-28

**Authors:** Yahya Thamrin, Dino Pisaniello, Cally Guerin, Paul Rothmore

**Affiliations:** 1 Department of Occupational Health and Safety, Faculty of Public Health, Hasanuddin University, Makassar 90245, Indonesia; 2 School of Public Health, University of Adelaide, Adelaide SA 5005, Australia; 3 School of Education, University of Adelaide, Adelaide SA 5005, Australia

**Keywords:** international students, university, worker, employment

## Abstract

International students represent an increasingly large segment of the Australian workforce. Most international students are working while studying, but there is a scarcity of quantitative data regarding potential work–study conflicts. Data from an online survey were analyzed with multivariate statistical methods to clarify the risk factors associated with perceived work–study conflicts in an Australian university. More than 66% of students felt that working demands interfered with their study. Negative impacts included tiredness and timetable clashes. Statistically significant correlates of work–study conflict were a perception of unfair wages and a lack of confidence in discussing occupational health and safety issues with employers. Underpayment may signify other vulnerabilities, such as unsafe working conditions. As many universities seek to increase their international student enrolments, these are important factors to consider for student retention. To mitigate this potential negative influence on study, universities should provide education and training related to international students’ rights and responsibilities in the workplace.

## 1. Introduction

The profile of international students in Australia is diverse and many factors may potentially influence their academic success and general experience in the Australian community. Factors that could be important include age and stage of life; whether they are alone or with family members; living and working arrangements; and the study program and its duration [1]. The study programs include exchange programs, English language courses, and higher education courses ranging from undergraduate degrees to masters and doctoral programs. International students in Australia come from all parts of the world, with the majority of students from China, India, and Malaysia [2]. Understanding the individual factors and interaction of factors is important for educational institutions and the community. Here we seek to explore the interaction between academic study and outside work.

The majority of international students work while studying. According to Australian Education International [3], about 56% of overseas students undertake paid jobs during academic periods. This increases to approximately 70% during university holidays. Similar patterns have been reported internationally. A survey of 20 UK universities reported that more than 50% of international students had undertaken paid work during their study with postgraduate students working more hours than undergraduate students [4].

International students can be characterized as young migrant workers [5]. As such, they frequently undertake jobs in the workplace that rank low in terms of employment status and skills [6,7]. Coupled with this, they are more susceptible to exploitation than local workers because they are often in need of additional income to support the financial burden of studying overseas [4,6].

Numerous studies have investigated international students’ working hours together with their rates of payment, including studies in Australia and New Zealand [6]. Their working conditions have been reported frequently in the media, confirming that many international students face exploitation and even discrimination in the workplace. However, most of those reports describe international students’ problems on a case-by-case basis [4].

### 1.1. International Students’ Employment Rights

Under the Migration Regulations 1994, international students have the right to work while studying in Australia for up to 20 h per week during the teaching periods and unlimited hours during study breaks [8]. These limitations are based on the assumption that the main objective of international students in Australia is to study rather than to work [9]. These limitations on hours of work do not apply to work or training that forms part of their course or for voluntary work—thus, it is only for paid employment. International students enrolled in a higher degree by research programs, and their family members, have permission to work unlimited hours once their research program has started [8].

### 1.2. International Students as a Working Population

Australia has a particular interest in recruiting international students as skilled migrant workers. The combination of an ageing population and low birth rates in Australia mean that young highly skilled people, who are just starting their careers, are needed for economic development.

In the United States, international students report their primary reasons for being there are: Obtaining academic achievement; gaining prestige as a US graduate; pursuing training and education not provided in their home countries; and escaping from unstable political and economic conditions in their home countries [10]. In contrast, most international students choose to study in Australia merely to obtain permanent residency through education [11,12]. However, international students tend to undertake low-level jobs while studying [4], therefore, this does not support their prospects of securing permanent residency because those jobs frequently bear no relation to their field of study.

A significant proportion of student migrant workers in Australia are employed in sectors such as agriculture and hospitality, which have reasonably high rates of accidents [13]. They face further difficulties in the workplace due to cultural adjustment and language constraints; they also report that some of their difficulties in the workplace are a result of racism and exploitation [4].

Together, these difficulties make international students a vulnerable workforce. However, it has been argued that the government and the trade unions have not taken a sufficient role in protecting these workers [6,14].

Regardless of these difficulties, most international students report feeling satisfied with their experience of working while studying. Hence, they prefer to stay in Australia after their graduation as migrant workers, and even recommend study in Australia to their friends and family [15].

Although there are some data on international students’ working hours and their rates of payment, there is a scarcity of research on the extent to which their work affects their ability to meet their study related demands and responsibilities—i.e., a work–study conflict, or interference. Research that does exist focuses on students in general, rather than international students specifically [16,17].

In this study, we explore factors associated with work-study conflict using an online survey of international students at the University of Adelaide.

## 2. Methods

### 2.1. Online Survey

An online survey of international students at the University of Adelaide was conducted using SurveyMonkey. The survey included the following categories (with example questions):

- Demographics—“What is your age?”; “What is your study program?”;
- Working experience—“What is your job status?”; “Do you think your wages are fair?”;
- Training experience—“Have you ever had any training in occupational health and safety (OHS)?”;
- Work–study conflict/interference—“Do you feel that your work interferes with your study?”;
- Role of the university—“Do you think the university has a responsibility to provide OHS education?”.

Questions relating to students’ OHS perception and confidence were included as an indicator of employee vulnerability.

With respect to work–study conflict/interference, the options were “never”, “sometimes”, and “often”. If “sometimes” or “often” the next question was how did it interfere? The options there include tiredness and timetable clash, with no further specification.

The survey questions were developed in conjunction with the International Student Centre (ISC) as there is no existing standardized questionnaire. The survey was piloted with a small number of international students (*n* = 23) for comprehension and logical flow. Invitations to complete the survey were emailed to all international students of the University by the International Student Centre (ISC). The survey invitations were sent in two waves. In the first wave, the invitation was sent through the international students’ email list with the subject heading “Invitation for survey for International Students.” The email included a survey information sheet, a consent form and the survey questionnaire. The second wave was sent three-weeks later as a reminder for all international students who had not joined the survey at the first invitation.

An online survey had three important advantages: It could reach all international students at the university; anonymity of respondents could be maintained; and the electronic interface allowed for accurate capture of the data.

Ethical approval for the collection on online survey data was provided by the University of Adelaide Human Research and Ethics Committee No. HS-2013.045.

### 2.2. Eligibiliy

Information about the survey indicated that it was only for enrolled international students who had experience of working while studying in Australia. Both undergraduate and postgraduate students were eligible.

### 2.3. Statistical Methods

The SurveyMonkey data were collected in Excel files, then cleaned and coded before analysis. Excel 2010 software (Microsoft, Washington, DC, USA)) was used to produce tables and graphs for the descriptive analysis and SPSS Version 21 (IBM, New York, NY, USA) was used to quantify the relationship between variables.

The primary outcome variable was whether or not there was a work–study conflict. Where this existed, the secondary outcome variables were how this interfered with their study in the form of tiredness or by creating a timetable clash. Bivariate and multivariate logistic regression analyses were conducted to investigate the association between independent variables and the outcome variables. Chi-square tests and odds ratio (OR) analyses were performed to see the association between variables. Statistical significance was defined as a two-tailed *p*-value of 0.05 or less. For each outcome variable, the statistically significant independent variables were included in a multivariate logistic regression model to identify important predictor factors.

## 3. Results

A total of 719 international students participated in the online survey, with 466 providing complete responses. Only complete responses were used in the analysis. There were some questions that participants were able to skip and some that allowed more than one option.

### 3.1. Online Survey—Descriptive Analysis

Information on participating student demographics, enrolment, financial support, reasons for working, rates of pay, and work-study conflict is presented in Table 1. The arithmetic mean age was 24.9 years with a median age of 24 years.

#### 3.1.1. International Students’ Reasons for Working

The three main reasons provided for working while studying were to get extra money (55.3%), to pay living costs (54.1%), and to supplement their living allowance (32.6%).

Interestingly, more than 30% of respondents revealed that one reason to be involved in the Australian workplace was to improve their English language skills while 28% of participants claimed that understanding Australian culture was their main motivation to take a paid job. The remaining reasons were to pay tuition fees (11.5%), for peer recognition (8.8%), and to support family in their home country (6.7%).

#### 3.1.2. Perception of Wages

A large percentage of respondents (42.7%) perceived that their wages were unfair.

#### 3.1.3. Hours of Work

Male students worked an average of 9.73 h per week in an area related to their field of study and 10.2 h per week in unrelated areas. Female students worked an average of 9.47 and 9.76 h, respectively.

#### 3.1.4. Work-Study Conflict

Almost 70% of participants reported that their employment created a work-study conflict.

#### 3.1.5. Outcome of the Work-Study Conflict

Among students who reported a work-study conflict, 234 (66.9%) reported this as causing tiredness, while 116 (33.1%) reported timetable/work clashes.

#### 3.1.6. Other Academic Factors

While not directly related to work-study conflict, 42 (13.7%) respondents reported that they had been unable to submit assignments on time, while 153 (50%) reported feeling stressed.

#### 3.1.7. OHS Perceptions and Confidence

A large percentage of respondents (61.4%) reported that they had received no OHS training and 42.3% reported they lacked the confidence to discuss OHS issues in the workplace. A majority (51.4%) of respondents considered that universities should provide education in OHS awareness.

### 3.2. Online Survey—Bivariate Analysis

Information on the relationship between the independent variables and the outcome variables is provided in Table 2.

#### 3.2.1. Work-Study Conflict

Positive associations were seen between the number of jobs (>1; OR 2.03, 95% CI 1.04–3.43) and a perception of wages as being unfair (OR 2.09, 95% CI 1.39–3.14). There was a negative association with being less confident about discussing OHS issues. Other variables were not statistically significantly correlated.

#### 3.2.2. Tiredness

There was a positive association between tiredness and being a second year student, with an unadjusted odds ratio (OR) of 1.8, 95% confidence interval (CI) 1.02–3.18). Positive associations were also seen for perceiving that wages were unfair (OR 1.89, 95% CI 1.31–2.75) and being less confident about OHS issues (OR 2.05, 95% CI 1.41–2.99).

However, negative associations were seen for being a study abroad or exchange program student (OR 0.35, 95% CI 0.18–0.67); being a PhD student (OR 0.41, 95% CI 0.22–0.78); and being an employee in the agricultural sector (OR 0.41, 95% CI 0.18–0.96).

#### 3.2.3. Timetable Clash

Positive associations were seen between timetable clash and being a partial scholarship student (OR 1.98, 95% CI 1.14–3.43); having a perception of unfair wages (OR 1.63, 95% CI 1.07–2.48); and working 20 h or more per week (OR 1.92, 95% CI 1.08–3.41).

### 3.3. Online Survey—Bivariate and Multivariate Analysis

Work–study conflict associated variables that were found to be significant in the bivariate analysis were included in a logistic regression model generating adjusted odds ratios (AOR). The AOR were used to identify important correlates of the outcome variables that can be seen in Table 2. For work-study conflict, significant associations remained for a perception that wages were unfair (AOR 2.11, 95% CI 1.39–3.19) and being less confident in OHS issues (AOR 0.49, 95% CI 0.33–0.73). For tiredness, significant associations remained for being a study abroad or exchange student (AOR 0.45, 95% CI 0.22–0.91) or PhD student (AOR 0.47, 95% CI 0.23–0.97); being less confident in OHS issues (AOR 1.72, 95% CI 1.16–2.55); and perceiving wages as unfair (AOR 1.64, 95% CI 1.10–2.46).

## 4. Discussion

Despite the positive effects for international students in Australia of having paid employment, such as improving language ability and cultural adjustment, it is important also to assess the potential negative ramifications of this work [18]. These impacts, which have been explored in several studies [19,20,21,22,23,24,25], include tiredness, stress, and an increased likelihood to withdraw from their study programs. Relatedly, many publications addressing international students’ working hours link exploitation in the workplace to a breach of visa entitlements [26,27].

Recent research reveals that many international students are involved in casual or part-time work and that they do so for numerous reasons including financial and social [28,29]. However, data from the International Student Barometer survey (ISB; 2013)—an independent survey conducted regularly at participating universities around the world—indicated that only about 9% of the students were spending more than 20 h per week in paid work; this number is even smaller when it comes to unpaid (voluntary) work. Even though this subgroup of students has the potential to breach their visa entitlement, they tend not to. The misconception of working too many hours may arise from statistics relating to international students who are in higher research degree programs (master or PhD by research) and who hold student visas that allow them to work unlimited hours once their program commences.

Recently, a substantial body of literature has revealed that international students are being exploited and underpaid in the Australian workplace [28,29]. In Adelaide, many overseas students were being paid below the minimum wage, working for only $6 per hour. When comparing the National Minimum Wage Order 2013 as a standard to be compared with the 2013 ISB data, it appears that more than 50% of international students claimed that they were being paid below the minimum wage. While we did not collect information on pay rates in our survey, respondents consistently reported a perception that their wages were unfair.

Reports of work–study conflict were significantly associated with a perception that wages were unfair and holding more than one job. While we did not explore this in our study, we postulate that it is likely that students receiving unfair wages may need to work at more than one job to provide a satisfactory income.

Interestingly, tiredness was negatively associated with being a PhD or study abroad student, meaning that they were less likely to be tired—a protective effect. Conversely, however, tiredness was positively associated with younger, undergraduate students. For PhD and study abroad students this negative association may be due to the fact that many of these students have a closer relationship with academic staff from whom they can seek advice and guidance than younger, undergraduate students do. However, the positive association seen with younger, undergraduate students may also be due to their inherent increased risk-taking behavior but we were unable to determine this.

Unfair wage perception was also significantly associated with tiredness, along with a reported lack of confidence in dealing with OHS issues. The perception of unfair wages reported by our respondents was also seen in the results from the ISB survey (2013) in which the students reported wages that were below the mandated national minimum. Such underpayment is a signal that other vulnerabilities, such as unsafe working conditions, are likely to accompany this type of exploitation [4,26,27]. We suggest that with more than 60% of our respondents reporting no OHS training and more than 40% indicating that they were not confident in dealing with OHS issues, this is a further example of the potential for workplace exploitation.

While working 20 or more hours per week was associated with timetable clashes in the bivariate analyses, this was not seen in the multivariate analyses. Nevertheless, this latter finding is consistent with Vickers, Lamb, and Hinkley [25] and Hovdhaugen [30] who found that the likelihood of students who work 20 h or more dropping out of tertiary education was between 160% to 200% greater than students who work less than 20 h per week.

This study provides valuable data for relevant stakeholders and policy makers to be more aware of young migrant workers’ problems and to develop strategies locally or nationally to improve students’ awareness of their rights and responsibilities. This is particularly important for a susceptible subgroup such as international students who represent a substantial proportion of young workers in Australia.

## 5. Limitations

This study also has some limitations. Firstly, the survey was conducted in only one university. However, the University of Adelaide is a typical metropolitan university of moderate size, thus this university is likely to be comparable with other Australian universities [31]. Hence, it can be argued that data and findings are likely to be more or less similar for other Australian universities. Secondly, the survey method employed one point of observation (that is, it was a cross-sectional study) where data on each participant was recorded once only. As a result, the associations between risk factors and outcome variables are not as strong as a cohort study could provide [32]. In addition, it was difficult to count the sample size from the real population based on email addresses. This is a limitation of online surveys [32]. Thirdly, some other potentially predictive factors such as social support and lack of freedom to choose to work that might be associated with work-study conflict have not been assessed [33]. Hence, a longitudinal study with a more comprehensive approach may provide a clearer indication of the associations between risk factors and outcome variables. Finally, although 719 international students commenced the survey, only 466 provided complete responses. This is for two reasons. Firstly, the online survey allowed participants to skip questions for which they were unwilling to share information. Secondly, although the online information indicated that the survey was only for those with working experience, international students could participate in the online survey whether they were working or not. For those who were not working it was not possible to complete all the questions. Hence, they were automatically excluded. However, we considered it unlikely that these factors significantly biased our results.

## 6. Conclusions

This research identified a perception of unfair wages and lacking confidence in OHS issues as correlates of work-study conflict among international students. As many universities seek to increase their international student enrolments, these are important factors to consider for student retention, particularly for undergraduate students.

As an education provider, and a part of their duty of care, universities should better prepare international students to work in the community and raise awareness of how working can interfere with study.

## Figures and Tables

**Table 1:** International student worker characteristics and experiences.

Variable	Number of Students	% of Total Respondents (*n* = 466)
**Student Demographics**		
Females	246	52.8
Married *	85	18.2
Have children *	53	11.4
Have family members with them *	100	21.5
**Academic factors**		
Faculty:		
Engineering, Computer, and Mathematics	124	26.6
Health Sciences	44	9.40
Humanities and Social Sciences	39	8.40
Sciences	75	16.1
The Professions	184	39.5
Study program:		
Undergraduate	171	36.7
Study Abroad or Exchange program	50	10.7
Master	190	40.8
PhD	55	11.8
Financial support:		
Private	323	69.3
Partial scholarship	31	6.70
Full scholarship	112	24.0
**Working experience**		
Job status (Casual/Seasonal)	208	44.6
Number of jobs (More than one)	56	12.0
Sector of industry:		
Restaurant	241	51.7
Supermarket/grocery/shop	120	25.8
Cleaning	72	15.4
Agriculture	33	7.1
Reasons for working *:		
To get extra money	275	55.3
To pay living costs	259	54.1
To supplement living allowance	156	32.6
To improve English language skills	146	30.5
To learn about Australian culture	134	28.0
To pay tuition fees	55	11.5
Peer recognition	42	8.8
To support family in home country)	32	6.7
Perception of wages (not fair) *	199	42.7
Working hours (20 or more)	60	12.9
Working conditions (outdoor)	102	21.9
New worker (working <1 year)	364	78.1
Not working under supervision *	142	30.5
**OHS training and experience**		
No OHS training *	286	61.4
Less confident in OHS issues *	197	42.7
University should provide OHS awareness and education *	194	51.4
**Work-study conflict**		
Work conflicts with study		
Never	157	33.9
Sometimes	277	59.8
Often	29	6.3
Outcome of work study conflict *		
Tiredness	234	66.9
Timetable clash	116	33.1
**Other academic factors**		
Failed to submit assignments on time *	42	13.7
Stressed *	153	50.0

* = Yes/No option.

**Table 2:** Work-study conflict, bivariate and multivariate analysis.

Variable	Primary Outcome Variable				Secondary Outcome Variables							
	Work-Study Conflict				Tiredness				Timetable Clash			
	Bivariate		Multivariate		Bivariate		Multivariate		Bivariate		Multivariate	
	OR	95% CI	AOR	95% CI	OR	95% CI	AOR	95% CI	OR	95% CI	AOR	95% CI
**Student demographics**												
Age (>20)	1.77	0.67–4.69			1.46	0.55–3.91			1.57	0.44–5.56		
Gender (being female)	0.84	0.57–1.23			1.17	0.81–1.68			0.72	0.47–1.09		
Marital status (being married)	0.66	0.41–1.08			0.76	0.48–1.22			0.49 *	0.26–0.92 *	0.56	0.28–1.11
Have children	0.80	0.44–1.45			1.03	0.58–1.84			0.50	0.23–1.09		
Have family members	0.81	0.51–1.28			1.01	0.65–1.58			0.64	0.37–1.11		
**Academic factors**												
Faculty:												
Engineering, Computer, and Mathematics	1.00				1.00				1.00			
Health Sciences	0.98	0.60–1.59			1.28	0.81–2.03			0.77	0.46–1.29		
Humanities and Social Sciences	0.74	0.36–1.55			0.58	0.30–1.15			1.22	0.55–2.74		
Sciences	0.99	0.48–2.06			0.64	0.32–1.30			1.05	0.46–2.38		
The Professions	1.35	0.77–2.36			1.23	0.72–2.11			0.96	0.53–1.87		
Year of study:												
1st year	1.00				1.00				1.00			
2nd year	1.23	0.68–2.22			1.8 *	1.02–3.18 *	1.92	0.99–3.75	1.05	0.54–2.03		
3rd year	0.88	0.48–1.60			1.46	0.83–2.57			0.69	0.36–1.31		
4th year	1.11	0.57–2.16			1.63	0.87–3.07			1.21	0.57–2.60		
Study program:												
Undergraduate	1.00				1.00				1.00			
Study abroad or exchange	0.33	0.17–0.61			0.35 *	0.18–0.67 *	0.45 *	0.22–0.91 *	0.60	0.27–1.33		
Master	0.51	0.23–1.11			0.57	0.26–1.27			0.89	0.32–2.46		
PhD	0.39	0.21–0.72			0.41 *	0.22–0.78 *	0.47 *	0.23–0.97 *	0.47	0.22–1.02		
Financial support:												
Private	1.00				1.00				1.00			
Partial scholarship	0.59	0.38–0.92			1.39	0.91–2.15			1.98 *	1.14–3.43 *	0.70	0.38–1.28
Full scholarship	0.98	0.44–2.20			1.56	0.74–3.28			2.73	0.93–8.02		
**Working experience**												
Job status (casual/seasonal)	0.74	0.51–1.10			0.89	0.62–1.28			0.62	0.45–1.07		
Number of jobs (>1)	2.03 *	1.04–3.96 *	1.90	0.95–3.79	1.37	0.78–2.41			1.12	0.59–2.11		
Industry sector:												
Restaurant	1.00				1.00				1.00			
Supermarket/grocery/shop	0.36	0.17–0.76			0.62	0.29–1.31			0.81	0.34–1.96		
Cleaning	0.37	0.17–0.81			0.79	0.36–1.74			0.74	0.29–1.87		
Agriculture	0.27	0.11–0.64			0.41 *	0.18–0.96 *	0.53	0.22–1.32	0.87	0.32–2.36		
Wage perception (not fair)	2.09 *	1.39–3.14 *	2.11 *	1.39–3.19 *	1.89 *	1.31–2.75 *	1.64 *	1.10–2.46 *	1.63 *	1.07–2.48 *	1.46	0.94–2.25
Working hours (20 or more)	1.48	0.81–2.72			1.35	0.78–2.33			1.92 *	1.08–3.41 *	1.79	0.99–3.23
Working condition (outdoor)	0.97	0.47–2.00			0.89	0.45–1.77			2.16	0.82–5.69		
New worker (<1 year)	0.91	0.57–1.46			0.83	0.53–1.28			0.96	0.58–1.59		
Not working under supervision	0.74	0.49–1.11			0.74	0.50–1.10			0.83	0.52–1.32		
**OHS training and experience**												
No OHS training	1.13	0.76–1.67			1.13	0.78–1.64			0.94	0.61–1.45		
Training not assessed	1.50	0.78–2.92			1.42	0.76–2.64			1.06	0.52–2.13		
Less confident in OHS issues	0.47 *	0.32–0.70 *	0.49 *	0.33–0.73 *	2.05 *	1.41–2.99 *	1.72 *	1.16–2.55 *	1.47	0.97–2.26		

* Statistically significant, *p* < 0.05; statistically significant for bivariate analysis; and statistically significant for multivariate analysis.
